# Transcriptome Analysis Reveals Early Hemocyte Responses upon In Vivo Stimulation with LPS in the Stick Insect *Bacillus rossius* (Rossi, 1788)

**DOI:** 10.3390/insects13070645

**Published:** 2022-07-18

**Authors:** Carlotta Bidoli, Andrea Miccoli, Francesco Buonocore, Anna Maria Fausto, Marco Gerdol, Simona Picchietti, Giuseppe Scapigliati

**Affiliations:** 1 Department of Life Sciences, University of Trieste, 34127 Trieste, Italy; carlotta.bidoli@phd.units.it (C.B.); mgerdol@units.it (M.G.); 2 Department for Innovation in Biological, Agro-Food and Forest Systems, University of Tuscia, 01100 Viterbo, Italy; fbuono@unitus.it (F.B.); fausto@unitus.it (A.M.F.); picchietti@unitus.it (S.P.); scapigg@unitus.it (G.S.)

**Keywords:** RNAseq, insect physiology, hemocytes, European stick insect, protease cascade, metabolic processes

## Abstract

**Simple Summary:**

Non-model insect species such as *B. rossius* suffer from a profound gap of knowledge regarding the temporal progression of physiological responses following the challenge with bacterial pathogens or cell wall components thereof. The reason for this mostly lies in the lack of genomic/transcriptomic resources, which would provide an unparalleled in-depth capacity in the analysis of molecular, biochemical, and metabolic mechanisms. We present a high-quality transcriptome obtained from high-coverage sequencing of hemocytes harvested from adult stick insect specimens both pre- and post-LPS stimulation. Such a resource served as the basis for a stringent differential gene expression and functional enrichment analyses, the results of which were characterized and discussed in depth. Selected transcripts encoding for C-type lectins and ML-domain containing proteins were further investigated from a phylogenetic perspective. Overall, these findings shed light on the physiological responses driven by a short-term LPS stimulation in the European stick insect.

**Abstract:**

Despite a growing number of non-model insect species is being investigated in recent years, a greater understanding of their physiology is prevented by the lack of genomic resources. This is the case of the common European stick insect *Bacillus rossius* (Rossi, 1788): in this species, some knowledge is available on hemocyte-related defenses, but little is known about the physiological changes occurring in response to natural or experimental challenges. Here, the transcriptional signatures of adult *B. rossius* hemocytes were investigated after a short-term (2 h) LPS stimulation in vivo: a total of 2191 differentially expressed genes, mostly involved in proteolysis and carbohydrate and lipid metabolic processes, were identified in the de novo assembled transcriptome and in-depth discussed. Overall, the significant modulation of immune signals—such as C-type lectins, ML domain-containing proteins, serpins, as well as Toll signaling-related molecules—provide novel information on the early progression of LPS-induced responses in *B. rossius*.

## 1. Introduction

Insects respond efficiently to the attacks of pathogens through potent innate immune responses that can be classified both as cellular and humoral [1]. The former are enacted by circulating hemocytes and include phagocytosis of bacteria, encapsulation of parasites, and secretion of soluble factors/mediators; the latter comprise melanization, coagulation, the production of reactive oxygen and nitrogen intermediates and the synthesis of antimicrobial peptides [2,3]. Melanization is a process that starts when pattern recognition receptors (PRRs) recognize the presence of specific pathogen-associated molecular patterns (PAMPs), such as bacterial lipopolysaccharide (LPS). This process leads to the activation of serine protease cascades and culminates with the conversion of the pro-phenoloxidase (proPO) zymogen in its functionally active form [4]. Hemolymph coagulation is a pathogen-induced process with striking analogies with blood clotting in vertebrates [5]. The magnitude and exact timing of all above-mentioned mechanisms vary greatly depending on the type of triggering factor [6].

The knowledge on insect immunity and, generally, physiology is mostly based on the studies carried out so far in *Drosophila melanogaster*. In recent years, data has been produced on a growing number of non-model insect species even by transcriptome analysis, but these aspects are largely unexplored in *Bacillus rossius* (Rossi, 1788) (Phasmatodea, Bacillidae), a common European stick insect [7]. Previous research conducted on this species has identified two main hemocyte subpopulations based on morphology and cytoskeletal patterns, namely plasmatocytes and granulocytes [8]; phagocytosis of bacteria, yeast particles, and latex beads was demonstrated in vivo and in vitro to be performed exclusively by plasmatocytes, likely due to their cytoskeletal features, as short as 1 h post-challenge [9]. *Bacillus* hemocytes were immunolocalized in situ using the anti-hemocyte monoclonal antibody (mAb) BrH1 [10] and quantified in the hemolymph of adult specimens by flow cytometry [11]. At the molecular level, however, no data exist on the modulation of transcriptional signatures in response to pro-inflammatory stimuli such as peptidoglycan (PGN) or LPS, which are commonly known to elicit different signaling pathways [12]. Such a gap of knowledge limits a deeper understanding of the functional biology of this stick insect species.

Building on the phagocytic properties of *B. rossius* hemocytes was demonstrated as short as 1 h post-treatment [9]; in this paper, we specifically set off to investigate the early stage transcriptional responses of hemocytes from unstimulated and in vivo LPS-stimulated *B. rossius* specimens to characterize the so far overlooked early response to bacterial stressors. LPS has already been employed for challenging several insect [13,14,15] or arthropod species [16,17] with the aim of elucidating immune signaling pathways; in some cases, similar short stimulation times were applied. A 2 × 60 M reads sequencing depth and a de novo assembly strategy were employed as the advances of RNA-seq have enabled the study of non-model insect species in a cost-effective and reproducible manner [18]. The functional annotation of strongly activated and differentially expressed genes (DEGs) in response to a short-term LPS stimulation highlighted a statistically significant enrichment of transcripts involved in proteolysis, carbohydrate and lipid metabolic processes, digestion, as well as the onset of immune-related processes (i.e., extracellular PRRs).

Taken together, our data represent a first step towards the elucidation of the LPS-driven molecular mechanisms involved in the early-stage hemocyte response of *B. rossius*, and provide a detailed in silico characterization of selected physiology-relevant genes in this species.

## 2. Results and Discussion

### 2.1. RNA-Seq Data Processing and Transcriptome Assembly

The RNA concentration and purity (A260/A280, A260/A230) for the pre- and post-LPS stimulation groups were 145.5 ng/μL, 1.867 and 1.824; and 27.3 ng/μL, 2.282 and 1.734, respectively.

A total of 239,468,810 and 241,299,648 raw reads were obtained from the sequencing of pre- and post-LPS stimulation samples.

The transcriptome was assembled de novo with 1,036,502,662 raw reads (480,768,458 from the present data and 555,734,204 from PRJNA578804 and PRJNA286345), equaling 69,100,177.47 reads per sample and an average length of 99.83 bp. After trimming, a total of 1,031,805,628 clean reads were generated, with a mean of 68,787,041.87 per sample and an average length of 99.83 bp. Detailed statistics per sample can be found in Table S1.

By exclusively maintaining the longest isoform per gene, the Trinity assembly yielded a transcriptome of 567,595 contigs. The number of contigs was reduced to 23,173 following filtering and reads decontamination (Table 1).

The transcriptome assembly was evaluated by BUSCO: 82.5% of genes in the Insecta Odb10 database were complete, 4% were present but fragmented, and 13.5% were missing.

Taking into account that several genes are expected to display a strong tissue specificity or to be strictly regulated, being either only expressed during early developmental stages or in response to specific stressors in adult individuals, these values altogether testify to the good quality of the obtained transcriptome (Table 1). A high-quality assembly was obtained by applying stringent filtering criteria, which excluded the effect of possible exogenous contaminants and removed a multitude of endogenous but poorly expressed transcripts. Nevertheless, because any of the discarded mRNAs reached a significant expression level neither in the pre- nor in the post-stimulation sample, the filtering process did not significantly impact the subsequent DEG analyses. Overall, the rate between complete and fragmented BUSCOs, equal to 20.6, points out that the overwhelming majority of the assembled transcripts likely included a complete ORF. The high quality and completeness of the assembled transcriptome was further evidenced by the presence of 2670 and 310 transcripts exceeding 5 and 10 Kb of length, respectively. The longest assembled contig measured 46,370 nucleotides.

Overall, 57.9% of the reads generated from the pre- and post-stimulation *B. rossius* hemocytes mapped to the reference transcriptome. Such a mapping rate was expected due to: i) the removal of contigs derived from mitochondrial and ribosomal RNAs, which can account for a significant fraction of all sequenced reads; and ii) the large number of contigs removed during the filtering process, denoting either poorly expressed transcripts or alternatively spliced mRNAs.

### 2.2. Functional Analysis of DEGs

**Figure 1:**
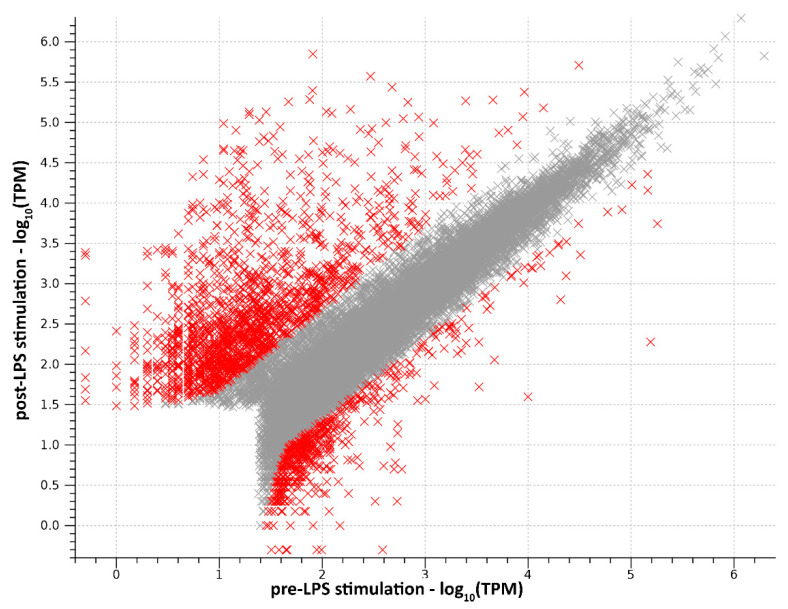
Scatter plot of log_10_-transformed gene expression levels observed in the pre- and post-LPS stimulated samples, calculated as TPMs. Differentially expressed genes are marked in red.

Differential expression analysis was performed by comparing the transcript abundance of hemocytes samples from post-stimulation vs. pre-stimulation insects. Overall, 2191 transcripts, corresponding to the 9.45% of the entire assembly, were found to be differentially expressed by more than |5| folds, indicating that the perturbation induced by LPS stimulation on the expression profile of hemocytes was highly significant even at early stages. The treatment effect was stronger on gene upregulation, as revealed by the presence of 1637 upregulated DEGs (7.06% of all assembled transcripts), whose median FC value was 16.29 X. On the other hand, 554 DEGs (2.39% of all assembled transcripts) were downregulated, with a median FC value equal to −8.31 X (Figure 1, Table S2a,b). A detailed in silico characterization and in-depth discussion of select genes and gene families is provided in the following sections.

### 2.3. A Subgroup of C-Type Lectins and ML Domain-Containing Proteins Were Strongly Upregulated within Two Hours of LPS Stimulation

The structural organization, functional role in innate immune response, and transcriptional regulation of insect C-type lectin (CTL) domain-containing proteins following stimulation have been broadly investigated in several insect species; however, to the best of our knowledge, no functional or structural information is presently available for CTLs in any species of the Phasmatodea order.

Most insect CTLs are established as strongly inducible secretory proteins produced in response to in vivo stimulations or challenges with pathogens [15,19,20]. CTLs act as PRRs in the extracellular environment; they recognize and bind to PAMPs via characteristic motifs localized within the CTL carbohydrate-recognition domain (CRD)—referred to as CLECT on SMART (accession number: SM00034)—overall playing an important role in both antiviral and antibacterial defenses [19,20]. In vertebrates, such motifs are conserved as Glu-Pro-Asn (EPN) or Gln-Pro-Asp (QPD) and are specific for mannose/glucose or galactose carbohydrates [21]. Insect CTLs, although with different binding specificity, can usually recognize a broad range of PAMPs, including PGN, LPS, and lipoteichoic acid (LTA) [22,23,24,25]. This allows the coating and agglutination of pathogens, which in turn stimulates their encapsulation and phagocytosis by hemocytes and triggers the nodulation process [25,26,27,28,29,30]. Moreover, some insect CTLs have been clearly implicated in the activation of the phenoloxidase cascade, leading to melanization [31,32,33]. *B. rossius* CTLs possessed both EPN and QPD motifs as well as NPE and NPV ones.

Most insect CRDs retain ligand binding capabilities although the corresponding motifs may be incomplete [34]: this suggests that the binding specificity of insect CTLs does not depend solely on the presence of EPN or QPD. Because it is shared by all insect sequences analyzed, the central proline residue may be fundamental for eliciting the recognition and binding functions typical of C-type lectins.

In general, insect CTL domain-containing proteins can be classified into three broad categories based on their domain architecture: (i) CTL-S, which have a simple structure with a single CRD; (ii) immulectins (IMLs), which include two tandem CRDs; and (iii) CTL-X, where one or more CTL domains can be combined with several other conserved domains in larger membrane-bound proteins [24,35,36,37].

**Figure 2:**
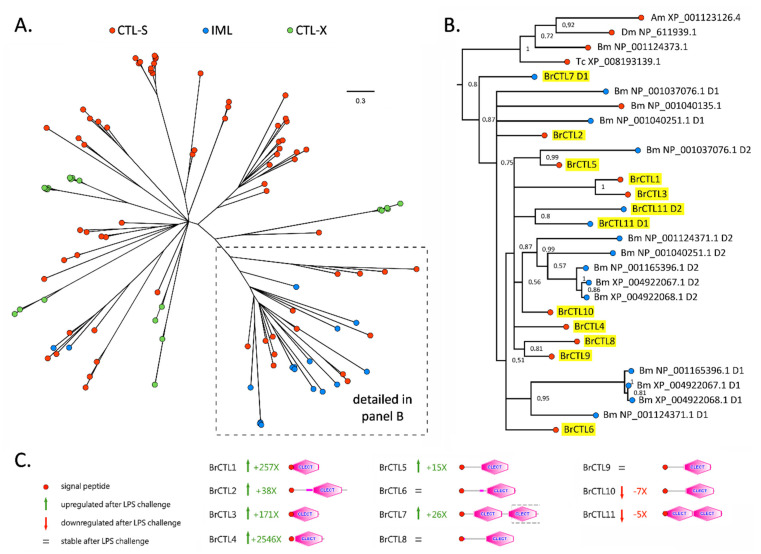
Bayesian phylogeny of insect CTL domain-containing proteins, including representatives from *Drosophila melanogaster* (Diptera), *Tribolium castaneum* (Coleoptera), *Apis mellifera* (Hymenoptera), and *Bombyx mori* (Lepidoptera), plus the 11 CTLs identified in *B. rossius*. The full unrooted tree, which depicts the complex evolutionary relationships among the three major structural classes of CTLs (i.e., CTL−S, IML, and CTL−X) is displayed in panel (**A**). The branch that includes all *B.* *rossius* CTLs (highlighted with a yellow background) are detailed in panel (**B**). The numbers shown close to each node represent posterior probability support values. Poorly supported nodes (i.e., <0.5) were collapsed. Dm: *D. melanogaster*; Am: *A. mellifera*; Tc: *T. castaneum*; Bm: *B. mori*. Panel (**C**) reports the domain architectures and gene expression trends following LPS stimulation of *B. rossius* CTLs. The portion of BrCTL7 included in a dashed box indicates a missing, unassembled region. D1 and D2 indicate the two CLECT domains of IMLs.

Based on their domain architecture, all the CTLs identified in the *B. rossius* hemocyte transcriptome belonged either to the CTL-S (nine sequences) or to the IML (two sequences) categories. The identification of IMLs in this species is worthy of note, as insect IMLs have been systematically described in Lepidopteran species (e.g., *M. sexta*) and, to the best of our knowledge, only in a handful of non-lepidopteran species. Although CTLs with two consecutive CRDs are fairly common among metazoan, their taxonomic distribution appears to be spotty in insects: for example, IMLs have been described in *Anopheles gambiae* (Diptera) [38] and *Tribolium castaneum* (Coleoptera) [39], but are absent in other Diptera (e.g., *Drosophila melanogaster*) and Hymenoptera (*Apis mellifera*). To date, IMLs have never described in Polyneoptera, so their identification in *B. rossius* represents the first report in Phasmatodea. While the size of some CTL-S proteins barely exceeded the length of the CTL domain (e.g., BrCTL3 was just 137 amino acids long), others included a low complexity N-terminal region of variable length. The two IMLs were, as expected, the longest CTLs, with BrCTL11 being 282 aa long and BrCTL7, the only incomplete CTL in *B. rossius*, likely exceeding 350 aa (Figure 2). Regardless of their domain organization, all stick insect CTLs displayed a well-recognizable signal peptide for secretion, indicating that these lectins are likely to exert their biological function in the extracellular environment.

Eight out of the 11 *B. rossius* CTLs were significantly differentially expressed following the LPS stimulation, including six CTL-S and two IMLs (Figure 2). Of these, six were upregulated and two were downregulated. Cumulatively, the expression of CTLs accounted for 0.06% and over 1.3% of the total transcriptional effort in the pre- and post-stimulation samples, respectively, resulting in an approximate 20-fold increase in expression. BrCTL1, BrCTL2 and BrCTL4 were mainly responsible for this trend, reaching ~3500, ~450, and ~9000 TPM, respectively, with extremely significant FC values following LPS stimulation: BrCTL4 was the fourth most highly expressed DEG in LPS-treated specimens; BrCTL1 was ranked 13th in the list of top 30 DEGs (Table 2); BrCTL2 was within the 300 most highly expressed mRNAs. The DEG condition of all 30 transcripts included in Table 2 was supported by the strongest statistics (i.e., Bonferroni-corrected *p*-value of 0). BrCTL3, BrCTL5, and BrCTL7 were also upregulated, even though their expression levels following LPS exposure were in all cases lower than 100 TPM. On the other hand, BrCTL11—and, particularly, BrCTL10—underwent significant downregulation, which accounted for ~83% of the total CTL expression pre-stimulation.

Previous comparative studies carried out in holometabolous insects have revealed that the types and number of CTL domain-containing proteins may largely vary among different species [35]. Moreover, CTL-S, IML, and CTL-X sequences do not belong to monophyletic clades [36,37], pointing out the dynamic evolutionary history of this gene family in insects, characterized by multiple independent CRD duplication events, as well as by its recruitment into complex multidomain proteins with various functions. The phylogenetic tree we obtained using representative sequences from Diptera, Hymenoptera, Lepidoptera, and Coleoptera (Figure 2A) was fully consistent with these observations.

Nevertheless, it is worth noting that all *B. rossius* CTLs were placed in the same branch of the CTL tree, together with proteins displaying both CTL-S and IML protein architectures (Figure 2B). Interestingly, in spite of their different domain organization, the stick insect sequences were placed with high posterior probability support (i.e., 0.87) with one CTL-S and several IML sequences from *B. mori*, even though no clear 1:1 orthology relationship could be established. This may suggest a functional convergence with the CTLs of Lepidoptera, which would most certainly deserve further investigation due to the significant divergence between this taxon of holometabolous insects and Phasmatodea. Although the evolutionary relationships among most *B. rossius* CTLs could not be fully resolved, the pairs BrCTL8/9 and BrCTL1/3 could be considered as paralogous genes, and the two domains found in BrCTL11 are most likely the product of a relatively recent CRD duplication event.

The MD-2-related lipid-recognition (ML) domain is ~150 aa/long and is found in single-copy in a variety of proteins such as myeloid differentiation 1 (MD-1), MD-2, GM2-activator (GM2A), Niemann-Pick C2 protein (Npc2), house-dust mite allergen proteins from *Dermatophagoides farinae* and *D. pteronyssinus*, and multiple proteins of unknown function in plants, animals, and fungi [40]. Although they also mediate chemical communication [41], the major roles of ML domain-containing proteins concern lipid metabolism, mainly through their lipid-binding properties, and immune response, by activating TLR-dependent intracellular signaling upon LPS binding [40]. In human, MD-2 is absolutely required for the TLR4 activation in the CD14/TLR4/MD-2 complex [42,43], which, when engaged by LPS, triggers the translocation of NF-κB transcription factors resulting in the production of pro-inflammatory mediators such as TNF, IL-1, and IL-6 [44]. In *M. sexta*, MLs directly bind to LPS via the lipid A moiety by means of a deep hydrophobic cavity sandwiched by the two anti-parallel β sheets [45]. In *Drosophila*, Npc2 proteins were demonstrated to also bind to additional bacterial components such as PGN and LTA. PGN, but not LPS, stimulates the activation of the diptericin promoter, suggesting that ML proteins may be involved in the immune deficiency (Imd) pathway leading to the expression of antimicrobial peptides [14]. At last, *Drosophila* and *Penaeus vannamei* ML protein-encoding transcripts were found overexpressed following LPS stimulation [14,16].

**Figure 3:**
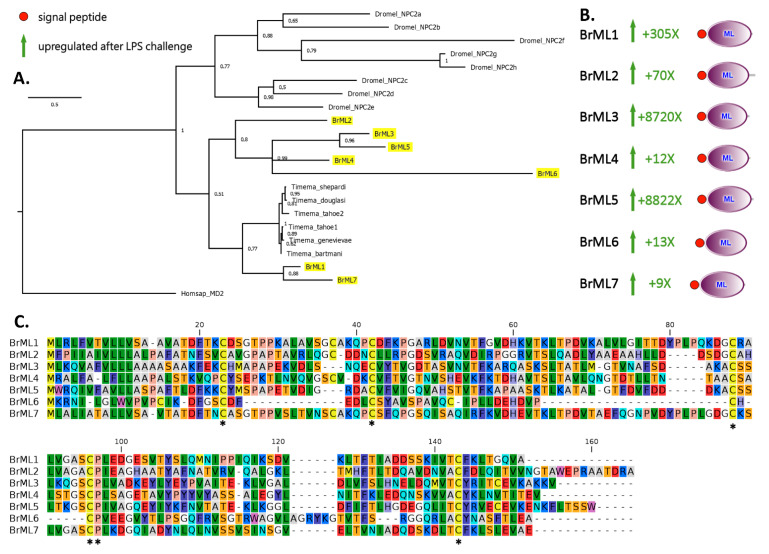
Bayesian phylogeny (**A**), domain architecture and gene expression trend following LPS stimulation (**B**), and multiple sequence alignment of *B. rossius* ML domain-containing proteins (**C**). The sequences from *B. rossius* are highlighted in yellow in the phylogenetic tree. Homsap: *Homo sapiens*; Dromel: *Drosophila melanogaster*; species name of the *Tinema* genus are expanded in the figure. The numbers shown close to each node represent posterior probability support values. Poorly supported nodes (i.e., <0.5) were collapsed. Asterisks indicate highly conserved residues in the MSA. The putative signal peptide of BrML6, which did not meet the significance threshold for being detected by SignalP, is indicated by a dashed circle.

ML domain-containing proteins were identified in a variety of invertebrate taxa (e.g., *Caenorhabditis elegans* [40]) and arthropod species (e.g., *D. melanogaster*, *Anopheles gambiae*, *Aedes aegypti*, *Tribolium castaneum*, *M. sexta*, *B. mori*, *P. vannamei*, and *Scylla paramamosain*) [14,16,46,47]. All possess a signal peptide and a domain with two pairs of conserved cysteine residues that enable the formation of disulfide bonds and, consequently, are fundamental for their tertiary structure. Seven transcripts encoding for ML proteins of 102–153 aa in length were characterized from the *B. rossius* transcriptome. Because the roles of most insect ML proteins are unknown, a classification based on the same criteria as in vertebrates was not possible and, for this reason, they were named BrML1–7. The protein structure deduced from BrML transcripts was consistent with that reported in literature for other insect ML-domain containing proteins, with the presence of a single ML domain, starting immediately after the signal peptide region. In spite of a variable pairwise sequence similarity, ranging from 17.2% (BrML3-BrML6) to 56.21% (MrML1-BrML7), all BrMLs displayed five conserved cysteine residues, as already reported in *M. sexta* ML-1 [45], and one conserved proline residue (Figure 3A). Compared to the other proteins, BrML6 lacked a detectable canonical signal peptide and displayed an unusual primary sequence, with a reduced loop between conserved cysteines no.2 and no.3 (Figure 3C).

Regardless of their structure, all BrMLs were heavily upregulated in their transcription in the early response to LPS stimulation, with FC values ranging between 8.53 (BrML7) and 88,221.77 (BrML5) (Figure 3B). Their absolute expression values were markedly different: BrML3 and BrML5 were ranked first (~25,400 TPM) and eight (~4600 TPM), respectively, among the most highly expressed DEGs in response to LPS stimulation (Table 2, Figure 3B); on the other hand, the five other BrMLs did not exceed 100 TPMs. Cumulatively, BrMLs accounted for nearly 3% of the total hemocyte transcriptional effort post-stimulation. Phylogenetic inference placed all the *B. rossius* ML proteins within a single monophyletic cluster, distinct from the one that included *D. melanogaster* NPC proteins, even though this group was not supported by high posterior probability (i.e., 0.51, Figure 3A). BrML1 and BrML7 were most closely related with the ML-domain containing proteins previously reported in various stick insects belonging to the genus *Timema*, whose function is currently unknown. The two most highly expressed and strongly upregulated ML proteins after LPS stimulation—i.e., BrML3 and BrML5—shared high similarity and should be considered as the product of paralogous genes.

### 2.4. Several Serine Proteases Are Strongly Overexpressed and May Trigger Melanization

At present, more than 1,100,000 proteases are available in release 12.3 of the MEROPS database of proteolytic enzymes and are grouped in approximately 4000 clusters of homologous sequences [46]. Even though the genomes of insect model species encode hundreds of serine proteases and homologous enzymes (e.g., more than 200 and 300 in the *D. melanogaster* and *A. gambiae* genomes, respectively) [47,48], the precise underlying biological pathways in which they are involved are still poorly understood. Serine proteases are key enzymes involved in numerous functions of physiological relevance, such as development, digestion, growth, molting, and immunity. In the context of immunity, they regulate innate responses such as melanization and the expression of antimicrobial peptides [49]. The melanization process—one of the most critical responses in insect immunity—can be elicited by multiple microbial cell wall components, including LPS [50,51]. The key molecular player in this process is phenoloxidase (PO), an enzyme that catalyzes the production of quinones for molecular cross-linking of melanin. PO is secreted as zymogen, prophenoloxidase (PPO), which shares remarkable sequence similarity with arthropod hemocyanins and is itself produced as an inactive proenzyme.

While multiple PPO genes are present in some insect species, we could unequivocally detect only a single PPO ortholog, named BrPPO (TRINITY_DN2649_c4_g1_i7) [52,53,54,55,56]. The longest ORF (1848 nt) encoded for a 615 aa sequence that shared a 58.74% and 56.01% similarity over a 99% coverage with PPOs of *D. melanogaster* (Q9V521.1) and *M. sexta* (O44249.3) in the swissprot database. The encoded protein sequence displayed the canonical domain architecture of other insect PPOs, with the hemocyanin N- and C-terminal conserved domains (PF03722 and PF03723) and a central tyrosinase domain (PF00264). BrPPO was one of the most abundant transcripts in *B. rossius* hemocytes of pre-treatment specimens (~30,000 TPM), indicating high constitutive expression in line with reported by Cerenius and Söderhäll [4]. BrPPO was not included in the list of DEGs and its expression following LPS treatment decreased to ~7000 TPM. The melanization process does not strictly require further transcriptional regulation of PPO itself, but can be also enacted through the upstream regulation of PPO activity by enzymatic cleavage from serine proteases [4]. Indeed, serine protease cascades are deeply involved in the whole melanization process: serine protease homologs (SPHs), containing a clip and a serine peptidase domain where glycine substituted the serine in the active site, are activated by uncharacterized proteases and in turn activate the so-called PPO activating factors (PPAFs), also known as PPO activating enzymes (PPAEs) or PPO-activating proteins (PAPs). Moreover, PPAFs—via downstream serine protease cascade—activate PPO, which is cleaved to yield active PO. Serine proteases display a similar domain architecture, which comprises one or more N-terminal CLIP domains (PF12032), combined with a C-terminal trypsin-like domain (PF00089) [57,58]. Two different sequences characterized by such a domain organization and showing significant homology with the PPAFs of *D. melanogaster*, *A. gambiae*, and *Holotrichia diomphalia* (i.e., TRINITY_DN104_c0_g1_i1, TRINITY_DN4815_c1_g3_i1) [57,58,59] were constitutively expressed in *B. rossius*.

The first serine proteases to trigger the melanization reaction are poorly characterized, but according to several studies they may involve initiator chymotrypsin-like peptidases [60], which may act as self-activating zymogens upon the binding with PAMP-complexed PRRs. Importantly, a relation between SPHs and LPS-binding IMLs was described in *M. sexta* [61], demonstrating how both PPAFs and PPO are recruited to the site of infection and how the PPO activation cascade is triggered by PRRs [49]. The activation of chymotrypsin-like peptidases may depend, in this case, on the binding of LPS by CTLs, as described in the previous sections. In this regard, a high number of trypsin-like serine proteases lacking CLIP domains and possibly initiating the serine protease cascade were strongly upregulated, as evidenced by the strong enrichment of the trypsin domain PF00089 among upregulated DEGs. These inducible serine proteases cumulatively accounted for nearly 4.6% of the total transcriptional effort in LPS-challenged hemocytes, but for just 0.025% in those of unchallenged stick insects. More specifically, we highlight that 14 and 21 genes encoding for chymotrypsin (TRINITY_DN39811_c0_g2_i1, TRINITY_DN12270_c0_g1_i1, TRINITY_DN129263_c0_g1_i1, TRINITY_DN1395_c0_g1_i10, TRINITY_DN16231_c1_g3_i3, TRINITY_DN17411_c2_g1_i1, TRINITY_DN236655_c0_g1_i1, TRINITY_DN31490_c0_g2_i3, TRINITY_DN33535_c0_g3_i1, TRINITY_DN6251_c1_g1_i2, TRINITY_DN712_c0_g1_i1, TRINITY_DN8207_c0_g1_i2, TRINITY_DN8207_c0_g2_i1, TRINITY_DN8207_c0_g3_i1) and trypsin (TRINITY_DN1061_c0_g1_i2, TRINITY_DN1061_c0_g2_i1, TRINITY_DN1061_c0_g3_i1, TRINITY_DN1061_c0_g4_i1, TRINITY_DN1238_c0_g1_i1, TRINITY_DN13980_c0_g1_i1, TRINITY_DN15507_c0_g1_i5, TRINITY_DN16_c1_g1_i3, TRINITY_DN16_c1_g4_i1, TRINITY_DN1852_c0_g1_i2, TRINITY_DN235704_c0_g1_i1, TRINITY_DN28212_c0_g2_i1, TRINITY_DN295861_c0_g1_i1, TRINITY_DN3815_c0_g1_i3, TRINITY_DN3847_c0_g1_i1, TRINITY_DN4089_c0_g1_i1, TRINITY_DN4089_c1_g1_i1, TRINITY_DN41541_c0_g1_i2, TRINITY_DN64224_c0_g2_i1, TRINITY_DN705_c2_g1_i1, TRINITY_DN705_c2_g2_i1) were identified as DEGs, with FCs ranging from ~8 to ~1270 and ~7 to ~5500, respectively. Of these, seven transcripts were identified within the top 30 DEGs (Table 2), cumulatively accounting for the 3.6% of the total transcriptional effort in hemocytes post-stimulation compared to the 0.003% of hemocytes pre-stimulation.

The remarkable upregulation of serine proteases may also suggest a different interpretation, taking into account their relevant sequence homology with a number of proteases with collagenolytic activities in crustaceans and insects [62,63,64,65]. It has been previously hypothesized that collagenase activity—though the generation of small collagen fragments—might stimulate immune response in *Galleria mellonella*, leading to the activation of Toll/Imd pathways, with the consequent nuclear import of Rel and production of AMPs [66].

In order to maintain homeostasis and avoid excessive inflammation, the serine-protease system is known to be negatively regulated by a family of serine-protease inhibitors called serpins. Previous studies have identified at least 12 families of serpins in insects—including *M. sexta*, *B. mori*, and *D. melanogaster* [67]—highlighting their key role in the regulation of immune response in insects. In particular, serpins affect the proPO activation in hemolymph by regulating the activation of the serine-protease cascade. We observed the expression of 17 transcripts containing the Serpin domain (Pfam: PF00079); in particular, five transcripts (TRINITY_DN3269_c1_g3_i3, TRINITY_DN542_c1_g1_i4, TRINITY_DN295931_c0_g1_i1, TRINITY_DN3269_c1_g4_i1, TRINITY_DN255859_c0_g1_i1) underwent a significant differential expression following LPS stimulation (Table S2a). Of these, four were upregulated and one (TRINITY_DN542_c1_g1_i4) was downregulated. Overall, this overexpression could explain the slight decrease in PPO observed after LPS stimulation.

### 2.5. Involvement of Toll and Imd Signaling Pathways in the Early Response to LPS

The activation of intracellular immune signaling cascades in response to bacterial challenges has been studied in detail in *D. melanogaster* and other model insect species. The Toll and Imd pathways are preferentially involved in the response towards Gram+ and Gram− bacteria, respectively [68], and regulate AMP production. LPS is a major component of the outer membrane of Gram− bacteria, but it does not play a significant role in the discrimination between Gram+ and Gram−bacteria in *Drosophila* [69,70]; rather, the activation of Imd is thought to be mostly dependent on the recognition of PGN by specialized peptidoglycan-recognition proteins (PGRPs) [71]. Still, LPS immunomodulatory activity is exerted through the activation of the PO-dependent melanization response [69]. On the other hand, it is presently unknown whether and to which extent the *Drosophila* immune system can be used as a model to infer the expected response of *B. rossius* to LPS: while vertebrate TLRs interact directly with LPS with the aid of LPS-bound lectins [72], leading to an activation of the intracellular signaling cascade in a Spätzle-independent manner [73], in *Drosophila* none of the nine Toll-like receptors act as PRRs [74].

As described in detail in the previous sections, the strong upregulation of different ML-domain containing proteins suggests that the ML protein/LPS complexes might be involved in triggering an intracellular immune signaling cascade culminating with the activation of NF-κB-like transcription factors upon direct binding with a TLR not orthologous to *Drosophila*’s Toll. Nevertheless, a parallel activation of the Toll signaling pathway via Spätzle mediation cannot be ruled out, since the Toll signaling and the melanization response in *Drosophila* can be activated by a shared cascade of extracellular serine proteases to amplify the initial invading signal and respond rapidly and efficiently to threats [67,75]. This involves specialized enzymes that share the same domain architecture of PPAFs and lead to the proteolytic cleavage of the cytokine Spätzle, which then dimerizes and acts as Toll ligand [76]. As per our results reported in the previous section, several serine proteases potentially involved in this process were strongly upregulated as early as 2 h post-challenge.

It is worth noting that the LPS used for in vivo stimulation of adult *B. rossius* specimens was purified by phenol extraction, with <3% protein impurities as determined by the Lowry method. Although it is possible that commercial LPS preparations be contaminated by PGN, at 2 h post-treatment we did not identify any differentially expressed gene encoding for proteins belonging to the PGRP or Gram− bacteria binding protein (GNBP) families, two well-known upstream regulators of the Toll and Imd pathways in *Drosophila* [77,78]. This is consistent with the expected role of such proteins in PGN, but not LPS, recognition, but evidence at longer LPS stimulation times must be gathered to rule out their involvement. Based on the transcriptional signatures found herein, the only plausible extracellular LPS sensors upregulated in *B. rossius* following a short-term stimulation are CTLs and ML-domain containing proteins. Both activate intracellular immune signaling through TLRs: the former through the binding between Toll and Spätzle (cleaved through a CTL/LPS-mediated serine protease cascade), and the latter though the binding between a TLR functionally analogous to the vertebrate TLR4 and ML-bound LPS. While, due to lack of evidence in literature, Imd involvement in LPS response appears unlikely, it needs to be remarked that in some insect species the functional boundary between Toll and Imd pathways is somewhat blurred due to significant crosstalk [79,80].

Both BrSpätzle (TRINITY_DN20_c0_g1_i6) and BrToll (TRINITY_DN27282_c0_g1_i1) could be identified with high confidence, and neither of them was differentially expressed. Four other TLRs were found in *B. rossius*: two of them, poorly expressed in hemocytes, displayed a significant homology with other characterized *Drosophila* TLRs, i.e., Toll-6/-7/-8, which are involved in morphogenesis and do not play a role in immunity [81]. The remaining two TLRs (TRINITY_DN316_c0_g1_i3, TRINITY_DN1459_c1_g2_i1) displayed poor homology with those of *Drosophila*, and may be therefore considered as the best candidates as interactors for the upregulated ML-domain containing proteins. One of these (TRINITY_DN27180_c0_g1_i2) was significantly upregulated in post-treatment hemocytes although at moderate expression level (i.e., TPM < 5), whereas the other (TRINITY_DN1359_c0_g2_i3) displayed a relatively high and stable expression (i.e., 103 TPM).

**Figure 4:**
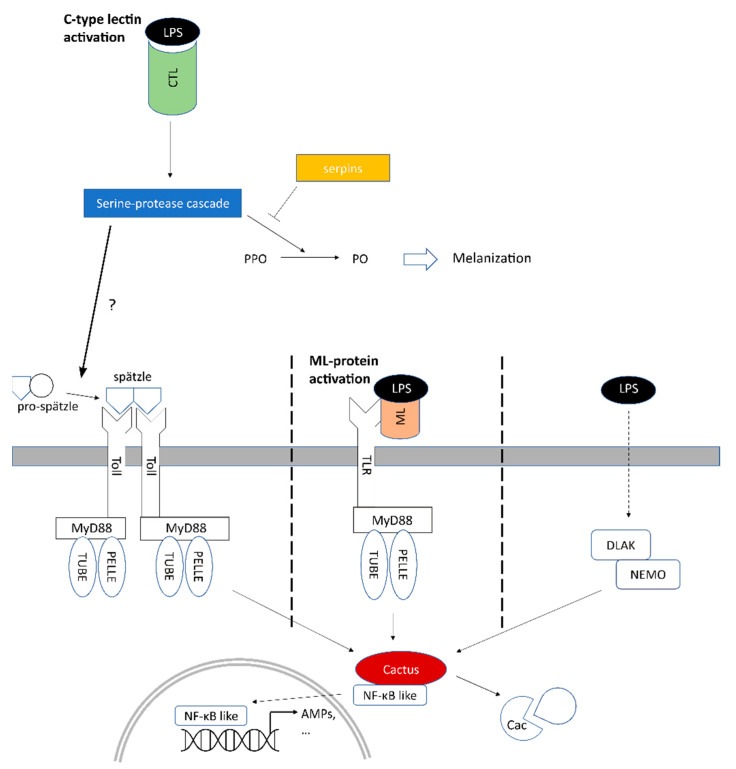
Schematic general overview of the immune pathways leading to the LPS-dependent modulation of NF-kB signaling in *B. rossius*. Colored background of shapes denotes modulation of molecules by the 2 h LPS stimulation (black).

In absence of a functional characterization, the involvement of the remaining cytosolic components of the Toll signaling pathway in *B. rossius* can be only speculated based on their homology with the well-characterized pathways of model organisms, such as *D. melanogaster*. Among these, we could detect with high confidence MYD88 (TRINITY_DN672_c3_g1_i2), Pelle (TRINITY_DN1057_c0_g1_i1), and Cactus (TRINITY_DN60558_c0_g1_i6) orthologs, plus a single transcript encoding for the ortholog to *Drosophila* Relish—a transcription factor belonging to the NF-kB family. Worthy of note, an interaction between IMLs and Relish was recently highlighted in *T. castaneum*, as an RNAi-mediated knockdown of the CTL under LPS stimulation resulted in the decrease in the transcription factor expression [15]. No sequence orthologous to Tube, a key kinase activated downstream of MYD88, could be detected [82]. None of the aforementioned molecular players was differentially expressed, with the exception of Cactus, the insect homolog of vertebrate IkB [83], and the target of phosphorylation by Pelle in *Drosophila*. The expression of this transcript increased by 11 folds following the 2 h LPS stimulation, reaching 133 TPMs. Cactus is a fundamental regulator of Toll signaling because, by binding to Dorsal or Dif in *Drosophila*, in a similar fashion to what IkB does with NF-kB in vertebrates, it prevents the nuclear import of such transcription factors. This process is reversed by the phosphorylation and consequent degradation of Cactus, mediated by Pelle [84]. The robust observed upregulation of Cactus at 2 h post-treatment is expected to result in the suppression of the NF-kB family-dependent expression of AMPs, which were in fact not detected as DEGs at such an early stage (Figure 4). On the other hand, the early physiological response we herein set off to investigate in *B. rossius* cannot exclude that the observed upregulation of Cactus is suppressed later in time, hence allowing for AMP production at a more advanced phase of the immune response.

DLAK/IKKβ is a kinase whose expression can be induced by LPS in *Drosophila* independently from Toll, and which can also phosphorylate, hence degrade, Cactus [85]. This kinase has been shown to associate with IKKγ/NEMO [86], and the combined action of these two factors regulates AMP transcription through Relish. At 2 h post-treatment, neither DLAK nor IKKγ orthologs were found to be differentially expressed in *B. rossius*.

In addition to the works of Scapigliati et al. of mid-1990s, only one study has so far attempted the characterization of the immune response of stick insects [87]. The repertoire of AMPs in the non-model species *Peruphasma schultei* was described via RNA-seq of whole insects at 24 h post-infection (time point selected based on peak induction kinetics) with a microbial elicitor mix composed of a heat-killed Gram+ bacteria and several yeast and bacterial cell wall components, including LPS at a concentration of 10 mg/mL. On one hand, the elicitor mix ensured the activation of a strong immune response in *P. schultei*; on the other, it compromised the identification of the individual contribution of each component to the immune response.

### 2.6. Upregulation of Other Immune Effectors and Processes

A few genes which likely encode proteins displaying a direct antibacterial activity were significantly upregulated following the short-term LPS stimulation.

Among these, it is worth mentioning the presence of four transcripts encoding proteins containing Insect allergen-related (PF06757 and PF16984) domains, and hereafter named IARPs. The four transcripts exceeded 100-fold inductions, reaching 5862 (BrIARP1—TRINITY_DN111798_c0_g1_i6), 2295 (BrIARP2—TRINITY_DN1615_c0_g1_i3), 1661 (BrIARP3—TRINITY_DN213_c0_g1_i1), and 2140 (BrIARP4—TRINITY_DN52225_c0_g2_i1) TPMs, respectively, being ranked well within the top 30 most highly expressed genes in the hemocytes of stimulated *B. rossius* (Table 2). BrIARP1-3 have similar size (208–212 aa) and structure, which comprises a signal peptide for secretion and a single IAR (PF06757) domain, but they display limited pairwise sequence similarity ranging from 26% to 38%. BrIARP4 is characterized by the presence of a structurally unrelated IAR domain (PF16984) [88].

Little information is available concerning the function of IARPs in insects, which in most cases concerns larger proteins, displaying several tandemly duplicated IAR domains. These proteins represent important allergens in humans [89,90,91] and are involved in detoxification processes in plant-feeding butterflies [92]. The function of single-domain IARs has long remained elusive, and limited to the *A. gambiae* G12 protein, over-expressed in the midgut following blood meal [93]. However, a recent study carried out in *A. aegypti* shed some light on their probable involvement in innate immune response too [94]. Thanks to its membranolytic properties linked with a lipid transfer mechanism, AEG12 can permeabilize and disrupt lipid-coated viruses and other lipid membranes. While the immune function of insect IARs has been so far only described in the context of antiviral defense, AEG12 has been shown to potentially act as a broad-spectrum lytic agent, which may therefore also target bacterial membranes. Therefore, we might speculate that BrIARs play a role in antibacterial defense in stick insects, being strongly upregulated in response to LPS stimulation.

A further gene whose product may be implicated in immune responses of *B. rossius* was found within the top 30 list of DEGs in Table 2 (i.e., TRINITY_DN12045_c0_g3_i2). Its expression underwent a 182 FC, going from ~8 TPM to 1334 TPM in unstimulated and stimulated insects, respectively. The corresponding protein was annotated with good confidence as calnexcitin-1: its structure comprises Ca^2+^-binding EFh domains (PF00036) and its localization was manually annotated in the endoplasmic reticulum (ER). Information on such protein is sparse: it was regarded as a protein implicated in associative learning in mollusks by means of ryanodine receptor activation [95] and K^+^ channel inhibition [96] or in feeding, mating, and reproduction in insects, where the gene is ubiquitously expressed in salivary glands and gonads [97]. Evidence exists about Ca^2+^-binding proteins being pivotal for phagocytosis in mammals, where they cause a cytosolic Ca^2+^ increase in the phagocytic cup of neutrophils [98] and are comprised in the phagosomes in macrophage-like cell lines [99]. Furthermore, cisternae of the ER were found closely associated with the phagosome membrane throughout the internalization process [100]. It is worth noting that if Ca^2+^ binding protein encoding genes are knocked out (i.e., calreticulin and calnexin), an arrest of phagocytosis at a stage preceding the extension of the phagocytic cup is observed [100] and clearance of *V. parahaemolyticus* is significantly decreased, pointing to the involvement of such proteins in innate immunity [101]. In addition, their transcription was positively stimulated by LPS as soon as 2 h post-challenge [101]. Given the shared expression site, location, and function, as well as the sharp transcriptional increase following LPS stimulation, we speculate that calnexcitin-1 is also involved in immune responses, possibly playing a role in phagocytosis. Scapigliati et al. [10], by means of the mAb BrH1, always immunolocalized *B. rossius* hemocytes close to injected stimuli, thus confirming their pivotal role in pathogen clearing.

**Figure 5:**
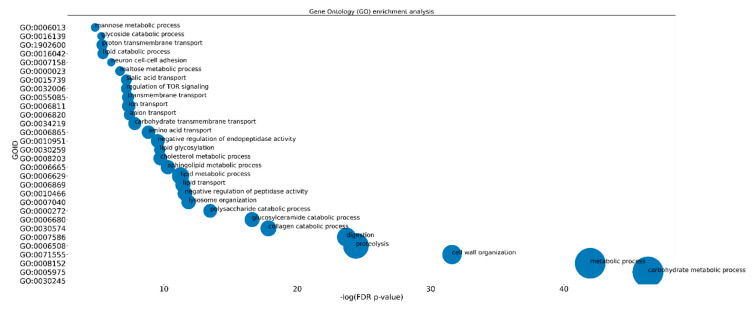
Scatterplot for significantly enriched Biological Process GO IDs against log10-transformed FDR-corrected *p*-values. Dot size is proportional to the number of DEGs mapping to that GO term. The reliability of statistical estimates increases from left to right along the *x*-axis.

Focusing on phagocytosis, pathogen internalization and destruction can only occur if biochemical modifications to the composition of phagosomes are precisely orchestrated along all steps of its maturation. Only the mature phagosome is degradative towards proteins, lipids, and carbohydrates thanks to the oxidative potential and the hydrolytic enzymes (e.g., proteases, lipases, nucleases, glycosidases, and phosphatases) acquired following the fusion with lysosomes and activated by a decrease in the pH to ~4.5 [102]. Leaving aside the already discussed abundance of peptidase-, G12-, and calexcitin-related transcripts, we call the attention on the fact that the top 30 list of DEGs was populated by hydrolytic enzymes (e.g., endoglucanase, lipase, polygalacturonase), with expression FC as high as ~6900 between experimental groups. Consistently, the most significantly enriched GO terms for biological processes were cellulose catabolic process (GO:0030245, FDR 0, 7 transcripts), carbohydrate metabolic process (GO:0005975, 1.21452E-49, 67), metabolic process (GO:0008152, 1.21994E-42, 68), cell wall organization (GO:0071555, 1.34655E-34, 23), digestion (GO:0007586, 7.05237E-25, 22), and proteolysis (GO:0006508, 2.59304E-23, 74) (Figure 5, Table S3a,b). 

Lipid transport, lysosome organization, and lipid metabolic process were overrepresented as well, overall testifying to the huge transcriptional effort being directed towards proteins underlying metabolic and catabolic processes- and, likely, phagocytosis-related processes in LPS-stimulated animals. Lipid metabolism was the second most represented pathway affected by DEGs following LPS stimulation in *Mytilus galloprovincialis* [103], even though the number of transcripts associated with such term was 1 order of magnitude lower compared to our results. In *B. rossius*, phagocytosis of foreign organisms or particles is enacted exclusively by plasmatocytes already after 1 h post-challenge with polystyrene latex particles or inactivated *E. coli* [9], with no information existing on LPS. On the other hand, LPS is effectively able to induce phagocytosis in crustaceans and insect hemocytes as soon as 1 h or 45 min post-stimulation, as demonstrated visually [103] and by gene expression analysis [104], respectively. Given the above-mentioned findings, the mass recruitment of digestive enzymes may sustain phagocytic activity in *B. rossius* following LPS stimulation. It is also possible that, along what is recognized as the invasion of a pathogen, cellular resources are allocated for sustaining the metabolically demanding processes enacted for its neutralization: changes in lipid metabolism (e.g., a shift from neutral lipid storage to phospholipid synthesis) were observed in *Drosophila* starting at 36 h post-infection with pathogenic *E. faecalis* [105], pointing to the intimate link between lipid metabolism and immune responses (i.e., immunometabolism) [106].

## 3. Materials and Methods

### 3.1. Insect Rearing, Experimental Stimulation, and Hemocyte Collection

Adult *B. rossius* were kept in containers under controlled conditions and fed with fresh bramble leaves throughout the experiment. 

Insects (n = 5) were challenged with ~50 μL/specimen of 200 μg/mL crude LPS preparation from *Escherichia coli* O55:B5 (Merck, catalog no. L2880, purity > 97%, phenol extraction purification method) by intra-abdominal injection, and hemolymph was collected 2 h later to obtain circulating hemocytes. Hemocytes pre-stimulation were collected 14 days earlier from the same specimens following the same procedure, except for LPS challenge. Hemolymph drops (50 to 70 μL/insect) were sampled by delicately puncturing the exoskeleton in the abdomen with a needle following sterilization with 70% ethanol and mixed with 0.5 mL PBS (0.15 M NaCl, 2.7 mM KCl, 1.5 mM KH_2_PO_4_, 8 mM Na_2_HPO_4_) pH 7.4 adjusted with 3 M NaCl to 350 mOsm Kg^−1^ and 10 mM EDTA to prevent coagulation. Hemocytes from both experimental groups were harvested at 500× *g* for 5 min, washed twice in PBS-EDTA, counted with a Neubauer chamber using 0.2% trypan blue in PBS to assess cell viability and resuspended in an appropriate volume of RNAzol^®^ RT (Merck, Darmstadt, Germany, catalog no. R4533). Following vortexing, homogenates were stored at −80 °C until processing.

### 3.2. RNA Extraction and Transcriptome Sequencing

Total RNA was extracted according to the manufacturer’s instructions using molecular grade reagents. RNA was eluted in 20 µL of RNAse-free water. RNA concentration and purity was determined spectrophotometrically (Picodrop Ltd., Hinxton, United Kingdom), while its integrity was verified by GelRed staining of ribosomal RNA bands on 1% agarose gel.

mRNA library preparation, carried out with an Illumina Total RNA Prep kit, and paired-end sequencing on an Illumina Novaseq 6000 platform with a 2 × 100 paired-end sequencing strategy and a depth of 2 × 60 M reads per sample, were outsourced to BMR Genomics (Padua, Italy).

### 3.3. RNAseq Data Processing—De Novo Assembly and Annotation

Raw reads, deposited into the NCBI SRA database under the BioProject accession no. PRJNA786449, were imported into the CLC Genomics Benchwork (Qiagen, Germany) and trimmed to remove residual adapters and low-quality nucleotides, with the base caller quality threshold set at 0.05 and reads shorter than 30 nt discarded. Trimmed reads were mapped to the mitochondrial genome of a closely related species (*Entoria okinawaensis*), available on NCBI, to remove mitochondrial sequences. Ribosomal sequences were removed by BLAST searches (E-value threshold < 1E-90) on a dataset of *B. rossius* ribosomal sequences available on NCBI. 

The transcriptome was assembled de novo using the TRINITY algorithm with default parameters using our present sequencing data and further RNAseq raw reads available at SRA—i.e., BioProject accession no. PRJNA578804 (gonads and legs from female *B. rossius*) and accession no. PRJNA286345 (generic sample from *B. rossius*). For redundancy reduction, only the longest isoform per gene was maintained and all contigs with length < 250 nt were removed. Transcriptome completeness was evaluated by Benchmarking Universal Single-Copy Orthologs (BUSCO) [107] using the Insecta OrthoDB10 database of orthologs.

Expression values for each contig were calculated by mapping the reads obtained within this work onto the assembled transcriptome using the CLC Genomics Workbench Mapping tool (Mismatch cost = 2; Insertion cost = 3; Deletion cost = 3; Length fraction = 0.95; Similarity fraction = 0.98; Global alignment = No; Strand specific = Both; Maximum number of hits for a read = 10). Contigs characterized by a very low expression level in both samples (i.e., Transcript Per Million (TPM) < 3) were discarded to remove possible exogenous contaminants. Annotation was performed with the Annotam pipeline (https://gitlab.com/54mu/annotaM, accessed on 30 June 2021); Gene Ontology annotations for each contig were integrated with the InterProScan algorithm. The resulting reference transcriptome was used for differential expression analysis.

### 3.4. DEG and Functional Enrichment Analysis

Due to the descriptive nature of the work and the availability of one pooled biological replicate per experimental group, the Differential Expression for RNA-Seq tool was conducted using the Kal et al.s’ test (*z*-test) on normalized gene expression data as input, assuming a negative binomial distribution. Each gene was modeled by a separate generalized linear model (GLM). Statistical stringency was applied to identify DE genes, using the following thresholds: *p*-value adjusted with the Bonferroni correction method < 0.01 and absolute fold change (|FC|) > 5.

Enrichment analyses of GO and Pfam terms within DEGs was performed using a hypergeometric test (one-sided Fisher’s exact test) setting a false discovery rate (FDR) *p*-value < 0.05 and number of DEGs (observed-expected) per each GO/Pfam term > 3.

### 3.5. Protein Prediction and Identification

Protein prediction was performed with Transdecoder v.5.5.0 (https://github.com/TransDecoder/TransDecoder/releases, accessed on 30 June 2021). The domain architecture was predicted using the simple modular architecture research tool SMART [108] (http://smart.embl-heidelberg.de/, accessed on 30 June 2021) and PFAM [109] (http://pfam.xfam.org/, accessed on 30 June 2021). Multiple sequence alignment (MSA) was achieved with MUSCLE [110] (https://www.ebi.ac.uk/Tools/msa/muscle/, accessed on 30 June 2021).

### 3.6. Phylogenetic Analysis

The regions corresponding to the C-type lectin (CTL) domains of the 11 *B. rossius* C-type lectins (BrCTLs) were fetched and aligned with a set of selected previously characterized insect CTLs [35] using Clustal Omega. In detail, *D. melanogaster*, *A. mellifera*, *B. mori*, and *T. castaneum* were selected as representative species for Diptera, Hymenoptera, Lepidoptera, and Coleoptera, respectively.

A similar approach was employed for the seven *B. rossius* ML-domain containing proteins. In this case, ML-domain containing proteins from different stick insect species belonging to the *Timema* genus, as well as the Npc2 proteins from *D. melanogaster* were retrieved from GenBank and added to the MSA dataset. The human MP-2 protein was used as an outgroup for tree rooting purposes.

The two MSAs were analyzed with Modeltest-NG [111] to identify the best-fitting model of molecular evolution, which was in both cases LG + I + G. The datasets were then analyzed with MrBayes v.3.2.7a [112] using two independent MCMC analyses run in parallel with four chains each for 1 million generations. Run convergence was checked with Tracer v.1.7 [113] and the phylogenetic trees were generated as majority rule consensus trees (i.e., nodes supported by posterior probability values < 0.5 were collapsed).

## 4. Conclusions

Knowledge on the temporal progression of immune defenses is key for understanding the pathways activated along the responses to pathogenic stressors. On the other hand, insights into changes in animal physiology *sensu lato* may help clarify or uncover the interplay between immunity and metabolic processes. The assembled transcriptome and the information herein reported lay the foundation for establishing the European stick insect *B. rossius* as a model organism and provide the genomic resources for future research on insect physiology.

Gene expression data were generated for stimulated and unstimulated conditions, allowing the identification of ~2200 differentially expressed genes among the 23,173 included in the refined de novo transcriptome assembly. The high stringency of the DGE analysis ensured the reliability of the results and excluded any influence of the experimental challenge method itself on the observed transcriptional modulation. An in-depth discussion was provided for several transcripts that are presumed to play an important role in the LPS-driven response and which displayed a highly significant modulation in their gene expression: among these were CTLs, MLs, serpins, as well as proteins involved in Toll- and Imd-related signaling (i.e., Spätzle, TLRs, Myd, Pelle, Cactus, Relish), IARPs, and calnexcitin-1. CTLs and MLs—the most likely LPS sensors in the extracellular environment—were further analyzed, providing an insight into their domain architectures and evolutionary relationships with other insect species. 

Overall, our data evidenced the complex molecular mechanisms underlying immune and metabolic responses enacted by *B. rossius* against a short-lasting stimulation with highly purified LPS. Combined with functional evidence to be obtained in vivo, they will be broadly interesting and relevant for supporting physiological studies and for successfully addressing evolutionary questions.

## Figures and Tables

**Table 1:** Summary statistics of the *B. rossius* de novo-assembled transcriptome with BUSCO completeness and integrity assessment against the Insecta Odb10 database.

Total TRINITY transcripts	23,173
Percent GC	41.41
Contig N50	3862
Median contig length	1572
Average contig	2319.10
Total assembled bases	53,740,459
Overall BUSCO score	C: 82.5% [S: 81.0%, D: 1.5%], F: 4.0%, M: 13.5%, n: 1367

**Table 2:** List of top 30 upregulated DEGs. Bonferroni-corrected *p*-value always equaled 0.

*Transcript ID*	*Annotation*	*Gene Expression Level (TPM)*		*Fold Change*
		Pre-LPS	Post-LPS	
TRINITY_DN276173_c2_g1_i1	BrML3	4.03	25,414.18	8720.33
TRINITY_DN8207_c0_g3_i1	Trypsin-like serine protease	11.47	12,709.61	1267.28
TRINITY_DN1061_c0_g3_i1	Trypsin-like serine protease	2.74	9178.71	3834.95
TRINITY_DN109048_c0_g2_i1	BrCTL4	4.04	9050.65	2545.93
TRINITY_DN5181_c8_g1_i1	Short secretory low complexity protein	14.32	7221.79	573.01
TRINITY_DN111798_c0_g1_i6	BrIARP1	9.12	5861.88	772.57
TRINITY_DN4089_c1_g1_i1	Trypsin-like serine protease	0.71	4683.20	4062.45
TRINITY_DN46586_c0_g2_i1	BrML5	0.32	4603.06	8821.77
TRINITY_DN1395_c0_g1_i10	Trypsin-like serine protease	66.67	4426.62	74.75
TRINITY_DN8856_c6_g1_i1	Lipoprotein lipase	3.98	4133.16	1243.97
TRINITY_DN4136_c0_g1_i8	Putative chitin-binding protein	36.08	4041.63	135.11
TRINITY_DN28212_c0_g2_i1	Trypsin-like serine protease	4.27	3707.48	1046.21
TRINITY_DN51244_c0_g1_i1	BrCTL1	16.60	3487.76	256.77
TRINITY_DN1757_c0_g1_i1	Lipoprotein lipase	0.82	3188.92	4361.47
TRINITY_DN317100_c0_g1_i1	Putative chitin-binding protein	8.84	2448.76	348.49
TRINITY_DN1615_c0_g1_i3	BrIARP2	17.81	2295.07	160.16
TRINITY_DN52225_c0_g2_i1	BrIARP4	9.51	2139.52	262.01
TRINITY_DN16_c1_g4_i1	Trypsin-like serine protease	0.31	1967.38	5513.76
TRINITY_DN994_c0_g2_i2	Endoglucanase	0.27	1958.19	6906.46
TRINITY_DN57351_c0_g1_i1	Lipoprotein lipase	28.59	1914.74	81.72
TRINITY_DN10752_c0_g1_i1	Fatty acid binding protein	177.40	1855.10	12.65
TRINITY_DN1099_c0_g3_i1	Uncharacterized protein	1.14	1848.74	1965.27
TRINITY_DN187_c0_g1_i1	Lipase	8.10	1800.15	276.31
TRINITY_DN1611_c0_g1_i1	Carboxylesterase	0.26	1790.37	6423.79
TRINITY_DN67078_c0_g1_i1	Lysosomal acid glucosylceramidase	0.92	1712.40	2258.41
TRINITY_DN213_c0_g1_i1	BrIARP3	14.21	1660.98	145.14
TRINITY_DN25071_c0_g1_i3	Zinc carboxypeptidase	26.19	1612.73	76.54
TRINITY_DN1061_c0_g1_i2	Trypsin-like serine protease	8.25	1610.60	239.02
TRINITY_DN950_c0_g6_i1	Zinc carboxypeptidase	27.03	1592.18	73.63
TRINITY_DN12045_c0_g3_i2	Calnexcitin-1	8.76	1334.26	181.60

## Data Availability

The data presented in this study are contained within the article or supplementary material. Raw reads are available from the NCBI SRA database under the BioProject accession no. PRJNA786449. The submission of the assembled transcriptome was successfully completed on NCBI TSA, and GJRR00000000 was assigned as accession no. The file will be publicly released to the TSA database at the time of publication.

## Supplementary Materials

The following supporting information can be downloaded at: https://www.mdpi.com/article/10.3390/insects13070645/s1, Table S1: Details on the number of raw and trimmed reads from samples sequenced in this work and from two BioProjects, nos. PRJNA578804 and PRJNA286345. The SRA experiment accession no. is indicated next to sample names; Table S2a: Upregulated DEGs (Fold Change > 5, Bonferroni *p*-value < 0.01). The “inf” annotation of selected genes is automatically assigned by the CLC Workbench “Create Fold Change Track” tool when one of the two expression values used for calculating fold changes values equals 0.; Table S2b: Downregulated DEGs (Fold Change < −5, Bonferroni *p*-value < 0.01). The “-inf” annotation of select genes is automatically assigned by the CLC Workbench “Create Fold Change Track” tool when one of the two expression values used for calculating fold changes values equals 0.; Table S3a: Significantly enriched Gene Ontology terms among upregulated DEGs; Table S3b: Significantly enriched Gene Ontology terms among downregulated DEGs.
