# Supplementary material for: Transcriptome Analysis Reveals Early Hemocyte Responses upon In Vivo Stimulation with LPS in the Stick Insect Bacillus rossius (Rossi, 1788)
Source: Insects. 2022 Jul 18;13(7):645. doi: 10.3390/insects13070645 (PMC9316843; doi:10.3390/insects13070645)
Supplement: Supplementary file 1 [file insects-13-00645-s001.zip › SupplementaryInformation_TableS1.pdf]

Supplementary information

Supplementary Table S1 - Details on the number of raw and trimmed reads from samples sequenced in this work and from two BioProjects # PRJNA578804 and PRJNA286345. The SRA experiment accession # is indicated next to sample names.

Sample	Raw reads	Trimmed reads
pre LPS_challenge (control)	239,468,810	237,703,224
post LPS-challenge	241,299,648	239,551,214
generic sample (SRR2230515)	14,863,824	14,853,454
gonads (SRR10323864)	43,650,866	43,568,100
legs (SRR10323863)	40,967,802	40,886,970
gonad (SRR10323862)	46,094,346	46,029,074
legs (SRR10323861)	34,612,676	34,553,254
gonad (SRR10323860)	44,731,242	44,641,708
legs (SRR10323859)	35,538,036	35,445,820
gonad (SRR10323858)	58,663,496	58,519,446
legs (SRR10323857)	42,283,932	42,171,556
gonad (SRR10323855)	48,824,438	48,733,278
legs (SRR10323854)	44,886,718	44,782,516
gonad (SRR10323853)	53,899,904	53,798,006
legs (SRR10323852)	46,716,924	46,568,008
Total	1,036,502,662	1,031,805,628
