# Supplementary material for: Transcriptome Analysis Reveals Early Hemocyte Responses upon In Vivo Stimulation with LPS in the Stick Insect Bacillus rossius (Rossi, 1788)
Source: Insects. 2022 Jul 18;13(7):645. doi: 10.3390/insects13070645 (PMC9316843; doi:10.3390/insects13070645)
Supplement: Supplementary file 1 [file insects-13-00645-s001.zip › SupplementaryInformation_TableS3a-S3b.pdf]

Supplementary information

Supplementary Table S3a - Significantly enriched Gene Ontology terms among up-regulated DEGs

Biological Process

GOID	FDR p-value	Term
GO:0030245	0	cellulose catabolic process
GO:0005975	1.214515580388E-49	carbohydrate metabolic process
GO:0008152	1.21993571077412E-42	metabolic process
GO:0071555	1.34655311301995E-34	cell wall organization
GO:0007586	7.05237265228835E-25	digestion
GO:0006508	2.59304219281037E-23	proteolysis
GO:0030574	3.7585767027274E-20	collagen catabolic process
GO:0006680	9.65791960050485E-19	glucosylceramide catabolic process
GO:0006869	1.59584502156171E-12	lipid transport
GO:0007040	1.64073717654072E-12	lysosome organization
GO:0006665	1.96961773792594E-12	sphingolipid metabolic process
GO:0006629	3.03546449835853E-12	lipid metabolic process
GO:0008203	8.699554686945E-12	cholesterol metabolic process
GO:0030259	2.96004131795191E-11	lipid glycosylation
GO:0010466	3.86133130302621E-11	negative regulation of peptidase activity
GO:0000272	3.08972841103233E-10	polysaccharide catabolic process
GO:0006865	9.49656688486223E-10	amino acid transport
GO:0010951	3.00103925560082E-09	negative regulation of endopeptidase activity
GO:0006820	4.66615053290409E-09	anion transport
GO:0015739	9.75904458530747E-09	sialic acid transport
GO:0032006	9.75904458530747E-09	regulation of TOR signaling
GO:0000023	4.30783666721812E-08	maltose metabolic process
GO:0007158	2.98135890193205E-07	neuron cell-cell adhesion
GO:0016139	1.69600790305773E-06	glycoside catabolic process
GO:1902600	2.25535325376343E-06	proton transmembrane transport
GO:0016042	3.2186291182804E-06	lipid catabolic process
GO:0006013	4.01712929751235E-06	mannose metabolic process
GO:0034219	6.09953064835086E-06	carbohydrate transmembrane transport
GO:0008202	8.32330046913199E-06	steroid metabolic process
GO:0019433	1.3692624940832E-05	triglyceride catabolic process
GO:0031333	1.3692624940832E-05	negative regulation of protein-containing complex assembly
GO:0006811	1.84349564963316E-05	ion transport
GO:0042742	4.94768611975568E-05	defense response to bacterium
GO:0050767	9.92534320191078E-05	regulation of neurogenesis
GO:0006633	0.000236102142452	fatty acid biosynthetic process
GO:0007602	0.00060245059553	phototransduction
GO:0007155	0.001415093794288	cell adhesion
GO:0006914	0.002064395452878	autophagy

Molecular Function

GOID	FDR p-value	Term
GO:0004650	0	polygalacturonase activity
GO:0008810	0	cellulase activity
GO:0030248	0	cellulose binding
GO:0045735	0	nutrient reservoir activity
GO:0016787	1.6374950118022E-74	hydrolase activity
GO:0016798	1.46714572931193E-70	hydrolase activity, acting on glycosyl bonds
GO:0008236	1.312572679975E-44	serine-type peptidase activity
GO:0004252	2.07812931328317E-38	serine-type endopeptidase activity
GO:0004553	8.00860808666312E-28	hydrolase activity, hydrolyzing O-glycosyl compounds
GO:0008233	8.70895876688556E-27	peptidase activity
GO:0052689	3.15491761195883E-25	carboxylic ester hydrolase activity
GO:0004348	1.70295508095181E-17	glucosylceramidase activity
GO:0008061	1.35544417589433E-16	chitin binding
GO:0080030	5.0093834081097E-14	methyl indole-3-acetate esterase activity
GO:0030414	1.26594203213536E-12	peptidase inhibitor activity
GO:0030246	6.64925433015046E-12	carbohydrate binding
GO:0046527	1.37405108648468E-10	glucosyltransferase activity
GO:0050295	1.37405108648468E-10	steryl-beta-glucosidase activity
GO:0004806	1.8265432874895E-10	triglyceride lipase activity
GO:0004867	3.16640751298529E-10	serine-type endopeptidase inhibitor activity
GO:0004180	6.43754918472335E-10	carboxypeptidase activity
GO:0015538	9.32070436590324E-10	sialic acid:proton symporter activity
GO:0005351	2.22967770349373E-09	carbohydrate:proton symporter activity
GO:0005319	2.24883179884189E-09	lipid transporter activity
GO:0004453	3.20488889123692E-09	juvenile-hormone esterase activity
GO:0015136	9.75904458530747E-09	sialic acid transmembrane transporter activity
GO:0004565	1.11433274064345E-08	beta-galactosidase activity
GO:0004185	4.30783666721812E-08	serine-type carboxypeptidase activity
GO:0004558	4.30783666721812E-08	alpha-1,4-glucosidase activity
GO:0032450	4.30783666721812E-08	maltose alpha-glucosidase activity
GO:0090599	1.13253996227628E-07	alpha-glucosidase activity
GO:0004465	2.98135890193205E-07	lipoprotein lipase activity
GO:0008237	3.94479865650776E-07	metallopeptidase activity
GO:0004177	1.4135023060096E-06	aminopeptidase activity
GO:0019137	1.69600790305773E-06	thioglucosidase activity
GO:0102799	1.69600790305773E-06	glucosinolate glucohydrolase activity
GO:0016298	1.85729486999473E-06	lipase activity
GO:0008239	4.01712929751235E-06	dipeptidyl-peptidase activity
GO:0008201	1.20014737464732E-05	heparin binding
GO:0005506	1.93874766847837E-05	iron ion binding
GO:0004620	2.21601897201465E-05	phospholipase activity
GO:0015293	2.68568643945578E-05	symporter activity
GO:0016491	9.76133762501589E-05	oxidoreductase activity
GO:0004181	0.000159412571869	metallocarboxypeptidase activity
GO:0004497	0.000337676502661	monooxygenase activity
GO:0008081	0.001339457644943	phosphoric diester hydrolase activity
GO:0016757	0.001743538174741	glycosyltransferase activity
GO:0008289	0.002268787371337	lipid binding

Cellular Component

GOID	FDR p-value	Term
GO:0005576	2.5098951236231E-109	extracellular region
GO:0005615	5.3958134550437E-32	extracellular space
GO:0005764	1.61564017209859E-27	lysosome
GO:0005765	6.6044097051477E-09	lysosomal membrane
GO:0009507	2.83986975603875E-07	chloroplast
GO:0009536	4.01712929751235E-06	plastid
GO:0062023	4.5775490831419E-06	collagen-containing extracellular matrix
GO:0005773	1.20014737464732E-05	vacuole
GO:0031225	3.45819480049203E-05	anchored component of membrane
GO:0005774	0.000471337134743	vacuolar membrane

Supplementary table S3b - Significantly enriched Gene Ontology terms among down-regulated DEGs

Biological Process

GOID	FDR p-value	Term
GO:0005576	1.30195504158305E-06	transmembrane transport
GO:0005886	8.83308292271826E-05	trehalose transport
GO:0045202	0.000446123597091	ion transport

Molecular Function

GOID	FDR p-value	Term
GO:0008061	2.35199582218479E-08	chitin binding
GO:0015574	8.83308292271826E-05	trehalose transmembrane transporter activity
GO:0051119	0.000128056436917	sugar transmembrane transporter activity
GO:0022857	0.000235074203931	transmembrane transporter activity

Cellular Component

GOID	FDR p-value	Term
GO:0005576	2.32786370003497E-08	extracellular region
GO:0005886	3.05222802041221E-08	plasma membrane
GO:0045202	0.000596750331085	synapse
